# Supplementary material for: Lifestyle and Psychological Factors Associated with Pregnancy Intentions: Findings from a Longitudinal Cohort Study of Australian Women
Source: Int J Environ Res Public Health. 2019 Dec 13;16(24):5094. doi: 10.3390/ijerph16245094 (PMC6950695; doi:10.3390/ijerph16245094)
Supplement: Supplementary file 1 [file ijerph-16-05094-s001.pdf]

Lifestyle and psychological factors associated with pregnancy intentions: Findings from a longitudinal cohort study of Australian women

SUPPLEMENTARY TABLES

Supplementary Table 1. Sensitivity analyses, reporting adjusted odds ratios (aOR), 95% Confidence Intervals (95%CI), and *p*-values from multivariable logistic regression analyses highlighting associations between pregnancy intentions and demographic, lifestyle and psychological variables at age 25 to 30 years (Wave 3).

	**Included women who were unable to have children**		**Included women or their partner who were unable to have children**	
**Variable**	**aOR (95%CI)**	***p*-value**	**aOR (95%CI)**	***p*-value**
Age	1.2 (1.1-1.3)	**<.001**	1.2 (1.1-1.3)	**<.001**
Number of children	0.5 (0.4-0.6)	**<.001**	0.5 (0.4-0.6)	**<.001**
Education				
No formal/high school	REF		REF	
Trade/diploma	0.9 (0.7-1.2)	.434	0.9 (0.7-1.2)	.437
Degree	0.5 (0.4-0.7)	**<.001**	0.5 (0.4-0.7)	**<.001**
Employment status				
No paid work	REF		REF	
Paid work	0.7 (0.5-1.0)	**.023**	0.7 (0.5-1.0)	**.031**
Annual household income (AUD$)				
<$25,999	REF		REF	
$26,000 - $77,999	1.0 (0.6-1.6)	.998	1.0 (0.6-1.6)	1.000
≥$78,000	0.9 (0.5-1.4)	.535	0.9 (0.5-1.4)	.547
Marital Status				
Not married or de facto	REF		REF	
Married or de facto	23.5 (12.7-43.2)	**<.001**	23.3 (12.7-42.9)	**<.001**
Country of birth				
Australia	REF		REF	
Other English-speaking background	0.9 (0.5-1.6)	.642	0.8 (0.5-1.6)	.591
Europe	0.3 (0.0-2.1)	.222	0.3 (0.0-2.2)	.226
Asia	1.2 (0.5-3.1)	.738	1.2 (0.5-3.1)	.730
Other	0.5 (0.1-4.1)	.538	0.5 (0.1-4.1)	.547
BMI Category				
Underweight	0.8 (0.4-1.5)	.542	0.8 (0.5-1.6)	.572
Normal weight	REF		REF	

Overweight	1.1 (0.9-1.5)	.317	1.1 (0.9-1.5)	.335
Obese	1.7 (1.3-2.3)	**<.001**	1.7 (1.3-2.3)	**<.001**
Physical activity				
Sedentary	REF		REF	
Low PA	0.9 (0.6-1.2)	.407	0.9 (0.6-1.3)	.415
Moderate PA	0.8 (0.5-1.2)	.315	0.8 (0.5-1.2)	.331
High PA	0.8 (0.5-1.2)	.355	0.8 (0.5-1.2)	.356
Sedentary behaviour (sitting time)	1.0 (0.9-1.0)	.317	1.0 (0.9-1.0)	.319
Diet quality	1.0 (1.0-1.0)	.930	1.0 (1.0-1.0)	.913
Alcohol intake				
None	REF		REF	
Any	0.7 (0.4-1.0)	**.037**	0.7 (0.4-1.0)	**.036**
Smoking				
Never or ex-smoker	REF		REF	
Current smoker	1.2 (0.9-1.5)	.224	1.2 (0.9-1.5)	.239
Depressive symptoms				
No	REF		REF	
Yes	0.9 (0.7-1.2)	.563	0.9 (0.7-1.2)	.574
Anxiety symptoms	0.9 (0.7-1.0)	.124	0.9 (0.7-1.0)	.139
Stress	1.1 (0.8-1.4)	.655	1.1 (0.8-1.4)	.637

Note. REF indicates reference category. Significant associations are indicated in **bold**. Multivariable analysis adjusted for all other variables in the table.

Supplementary Table 2. Sensitivity analyses, reporting adjusted odds ratios (aOR), 95% Confidence Intervals (95%CI), and *p*-values from multivariable logistic regression analyses highlighting associations between pregnancy intentions and demographic, lifestyle and psychological variables at age 31 to 36 years (Wave 5).

	**Included women who were unable to have children**		**Included women or their partner who were unable to have children**	
**Variable**	**aOR (95%CI)**	***p*-value**	**aOR (95%CI)**	***p*-value**
Age	1.0 (1.0-1.1)	.493	1.1 (1.0-1.1)	.096
Number of children	0.4 (0.3-0.5)	**<.001**	0.4 (0.3-0.4)	**<.001**
Education				
No formal/high school	REF		REF	
Trade/diploma	1.2 (0.8-1.6)	.385	1.1 (0.8-1.4)	.820
Degree	1.0 (0.7-1.4)	.886	0.8 (0.6-1.1)	.127
Employment status				
No paid work	REF		REF	
Paid work	0.6 (0.5-0.8)	**.001**	0.8 (0.6-1.0)	.106
Annual household income (AUD$)				
<$25,999	REF		REF	
$26,000 - $77,999	1.0 (0.4-2.0)	.939	1.0 (0.5-1.8)	.924
≥$78,000	1.1 (0.5-2.2)	.847	1.0 (0.5-1.9)	.995
Marital Status				
Not married or de facto	REF		REF	
Married or de facto	21.5 (12.8-36.4)	**<.001**	21.2 (12.6-35.6)	**<.001**
Country of birth				
Australia	REF		REF	
Other English-speaking background	1.1 (0.6-1.8)	.781	1.1 (0.7-1.8)	.586
Europe	1.6 (0.7-3.7)	.264	1.6 (0.7-3.7)	.267
Asia	0.5 (0.2-1.6)	.268	0.5 (0.2-1.3)	.143
Other	1.2 (0.2-5.6)	.837	0.9 (0.2-4.0)	.874
BMI Category				
Underweight	0.8 (0.4-1.7)	.597	0.9 (0.5-1.7)	.739
Normal weight	REF		REF	

Overweight	0.8 (0.4-1.6)	.517	0.8 (0.4-1.6)	.577
Obese	1.1 (0.5-2.3)	.779	1.2 (0.6-2.2)	.670
Physical activity				
Sedentary	REF		REF	
Low PA	1.1 (0.8-1.7)	.500	1.0 (0.7-1.4)	.923
Moderate PA	1.0 (0.7-1.5)	.930	1.1 (0.8-1.6)	.648
High PA	0.8 (0.5-1.2)	.323	0.8 (0.6-1.2)	.314
Sedentary behaviour (sitting time)	1.0 (0.9-1.0)	.071	1.0 (0.9-1.0)	.129
Diet quality	1.0 (1.0-1.0)	.586	1.0 (1.0-1.0)	.433
Alcohol intake				
None	REF		REF	
Any	0.7 (0.5-1.1)	.156	0.8 (0.6-1.2)	.249
Smoking				
Never or ex-smoker	REF		REF	
Current smoker	0.8 (0.5-1.1)	.097	0.8 (0.6-1.1)	.120
Depressive symptoms				
No	REF		REF	
Yes	1.1 (0.8-1.5)	.472	1.0 (0.8-1.3)	.998
Anxiety symptoms	0.8 (0.8-1.1)	.192	0.9 (0.8-1.1)	.367
Stress	0.9 (0.7-1.1)	.274	0.9 (0.8-1.2)	.613

Note. REF indicates reference category. Significant associations are indicated in **bold**. Multivariable analysis adjusted for all other variables in the table.
